# A validation study regarding a generative approach in choosing appropriate colors for impaired users

**DOI:** 10.1186/s40064-016-2659-6

**Published:** 2016-07-15

**Authors:** Luigi Troiano, Cosimo Birtolo, Roberto Armenise

**Affiliations:** Department of Engineering, University of Sannio, Viale Traiano, 82100 Benevento, Italy; Poste Italiane S.p.A., P.zza Matteotti 3, 80133 Napoli, Italy

**Keywords:** Accessibility, User interface design, Dichromacy, Color vision deficiency, Interactive genetic algorithms, Color perception, Search based software engineering

## Abstract

In many circumstances, concepts, ideas and emotions are mainly conveyed by colors. Color vision disorders can heavily limit the user experience in accessing Information Society. Therefore, color vision impairments should be taken into account in order to make information and services accessible to a broader audience. The task is not easy for designers that generally are not affected by any color vision disorder. In any case, the design of accessible user interfaces should not lead to to boring color schemes. The selection of appealing and harmonic color combinations should be preserved. In past research we investigated a generative approach led by evolutionary computing in supporting interface designers to make colors accessible to impaired users. This approach has also been followed by other authors. The contribution of this paper is to provide an experimental validation to the claim that this approach is actually beneficial to designers and users.

## Background

Colors can be one of the main barriers to the Information Society for color vision impaired users.^1^ Colors convey concepts, ideas and emotions, according to cultural and individual traits. If there exist colors entailing joy and relax, others produce excitement or melancholy. Therefore, not perceiving the colors properly can invalidate the purpose of communication.

In 2004, the UK Disability Rights Commission (DRC)^2^ reported their formal investigation regarding accessibility of websites in the UK (Disability Rights Commission 2004). Surprisingly, the 81 % of sample did not meet W3C basic guidelines for accessibility, although most of website designers and owners are aware of the importance of making accessible the Web. According to this study, misperceived colors are the second main barrier to impaired users. In order to break down this barrier, there is a shared understanding for the need of a set of the best practices and guidelines in designing user interfaces (e.g., see Wang et al. 2010; Nguyen et al. 2014).

The W3C has been largely working on this topic and proposed a set of recommendations (known as Web Content Accessible Guidelines, WCAG) aimed at making the Web accessible to users with impairments, visual among them. WCAG 2.0 (WCAG 2008) has been published in December 2008. Guidelines state four principles that provide the foundations of Web accessibility:

- *Perceivable* “Information and user interface components must be presentable to users in ways they can perceive.”
- *Operable* “User interface components and navigation must be operable.”
- *Understandable* “Information and the operation of user interface must be understandable.”
- *Robust* “Content must be robust enough that it can be interpreted reliably by a wide variety of user agents, including assistive technologies.”

The WCAG defines a 3-level scale (Level A, Level AA and Level AAA) in assessing interface accessibility. WCAG underlines some important intents related to criteria above. The first (Level A) is that colors are an important asset in web pages, as chromatic differences may convey information and each color may have a meaning assigned to it. For instance, fields could be marked by red boxes when wrong input is given, or values in a table could be highlighted by green while others by red. The other two (Levels AA and AAA) take into account that hue and saturation generally do not affect legibility in able-bodied users (Knoblauch et al. 1991). Differently color deficiencies can affect how luminance contrast is perceived. Although ISO provides 3:1 as minimal ratio threshold for standard color vision, WCAG suggests to use respectively 4.5:1 and 7:1 for Level AA and Level AAA.

In accordance to WCAG and DRC recommendations, Stanca Act (approved by Parliament of Italy in 2004) also states that contrast between background and foreground is one of the key factor in making the Web accessible for all. The importance of taking into account color usability and accessibility guidelines has been reaffirmed by Webster (2014) in a recent article appeared in the ACM Interactions magazine.

Accessible color schemes guarantee high luminance contrast between colors (e.g. foreground and background) not only when they are perceived by able-bodied users, but also by those with vision disorders. Accessibility apart, color selection still attains to artistic abilities and aesthetics. Therefore, preservation of original UI chromatic choices, some of them related to the meaning of colors, should be important as much as enabling UI to the broader audience made of users affected by color vision deficiency (CVD).

However, attempting to fulfill both needs can often result into conflicts, solving them generally means to experiment several combinations, thus looking for an acceptable trade-off between different options. Searching for a satisfying color palette can be regarded as a problem of combinatorics. In order to test if a particular combination of colors is accessible, a useful technique, according to DRC suggestions, is to convert a web page to black and white and to verify if all information is properly conveyed. This method is unfeasible when exploring a large number of alternatives. A different way to test accessibility is to simulate how colors are perceived by users affected by some kind of color vision deficiency, and to check if a proper contrast ratio is reached according to WCAG definition. This approach makes possible to explore a wider number of possible alternatives by search algorithms. In particular genetic algorithms can offer a reliable means to search the color space, exploiting those solutions that exhibit a good fit to the set of requirements.

In the past, we experimented the application of GA to support different tasks concerning the UI design (Armenise et al. 2010; Filipe and Cordeiro 2010; Birtolo et al. 2010; Troiano et al. 2009a, b; Russo et al. 2008; Troiano et al. 2008a, 2015). Among them we considered the problem of adapting color schemes to users affected by vision disorders by means of standard genetic algorithms (SGA) (Troiano et al. 2008b) and interactive genetic algorithms (IGA) (Birtolo et al. 2009) (more details are contained in Troiano and Birtolo 2014).

In this paper we will finally attempt to answer to the question if there is evidence that UI design can benefit of support provided by interactive and non-interactive genetic algorithms. As case studies we will focus on Protanopia (incidence 1.3 % males, 0.02 % females) and Deuteranopia (incidence 1.2 % males, 0.01 % females) due to their severity, but the approach being presented can be easily extended to other disorders. The study is made of two experiments. The first is aimed at experimenting how effectively GA can support website and user interface designers in choosing a combination of colors (i.e.,color palettes) that moving from an initial choice is able to evolve towards alternatives that, if the one side are accessible to broader users, on the other side they still preserve the designer’s preferences, which can be made explicit or implicit. The second experiment is aimed at testing if a solution generated by this kind of support is competitive to human-generated color schemes in terms of usability for CVD users. The remainder of this paper is structured as follows: Section 2 overviews how color vision can be modeled for those readers that are not familiar with, and outlines related works and contributions to the problem of adapting colors to CVD users as search; Section 3 provides experimental settings and outcomes; Section 4 discusses conclusions and possible future directions; “Appendix” offers an overview of the field.

## Color vision, deficiencies and adaptation

**Fig. 1:**
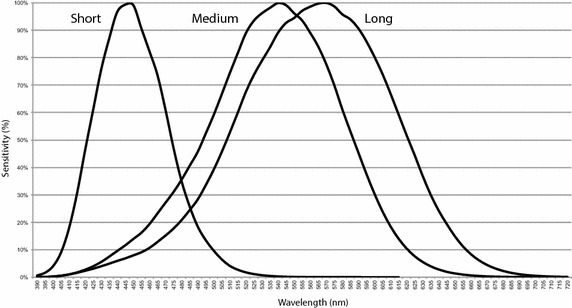
Spectral sensitivity curves of L, M and S cones

Human vision is generally trichromatic. Colors are perceived by three classes of cells, called *cones*, able to absorb photons by different sensitivity with respect to the light wavelength. When the peak sensitivity lies in the long-wavelength of the visible spectrum (560–580 nm) we have cones of type L, in the middle-wavelength (530–540 nm) we have M cones, and in the short-wavelength (420–440 nm) there are S cones. The standard spectral sensitivity is reported in Fig. 1, where cone sensitivity is plotted at different wavelengths according to Stockman and Sharpe (2000).^3^

Dysfunction of cones (e.g., resulting in spectral sensitivity peak shift or shape alteration) can heavily affect the way color are perceived. Color vision deficiency has place when cone cells exhibit a partial or complete loss of function. The main types of CVD are known as *anomalous trichromatism*, *dichromatism* and *monochromatism*.

Anomalous trichromatism is due to a sensitivity peak shift of one of the fundamental cones. This deficiency is further classified in *protanomaly*, when malfunction is given by L cones, and *deuteranomaly*, when the disorder is associated to M cones. Depending on the extent the peak is shifted, the perception of anomalous trichromats can range from almost normal to dichromatic vision. Indeed, it has been estimated (DeMarco et al. 1992) that on average the peaks are respectively at 440, 543 and 566 nm for standard average vision, at 440, 543 and 553 for *protanomalous trichromats*, while *deuteranomalous trichromats* show peaks at 440, 560 and 566 nm. Therefore, vision deficiency is due to the lower distance between L and M sensitivity peaks (i.e.,respectively 10 and 6 nm).

When distance is reset to zero, one of the fundamental cones is missing, resulting into a severe form of CVD known as *dichromatism*. Therfore, dichromats confuse colors only in the spectrum range of the missing photopigments, as they lack one class of cones. In particular, dichromatism is divided in as *protanopia*, *deuteranopia* and *tritanopia*, depending on which cones among L, M or S are missing. The most common forms of CVD are listed in Table 1, as reported by the MPEG-21 standard specification (Yang et al. 2004).

**Table 1:** Medical term and CVD descriptions in the MPEG-21 (Yang et al. 2004)

Medical term	Deficiency type	Deficiency degree
Protanomaly	Some reduction in discrimination of the reddish and greenish contents of colors, with reddish color appearing dimmer than normal	Mild
Protanopia	Severely reduced discrimination of the reddish and greenish contents of colors, with reddish color appearing dimmer than normal	Severe
Deuteranomaly	Some reduction in discrimination of the reddish and greenish contents of colors	Mild
Deuteranopia	Severely reduced discrimination of the reddish and greenish contents of colors	Severe
Tritanomaly	Some reduction in discrimination of the bluish and yellowish contents of colors	Mild
Tritanopy	Severely reduced discrimination of the bluish and yellowish contents of colors	Severe
Incomplete achromatopsia	Deficiency in both L cone sensitivity and M cone sensitivity. No color discrimination, and there is approximatively normal brightness of colors	Mild
Complete achromatopsia	Deficiency L cone sensitivity, M cone sensitivity and S cone sensitivity. No color discrimination, and brightness is typical of scotopic vision	Severe

**Fig. 2:**
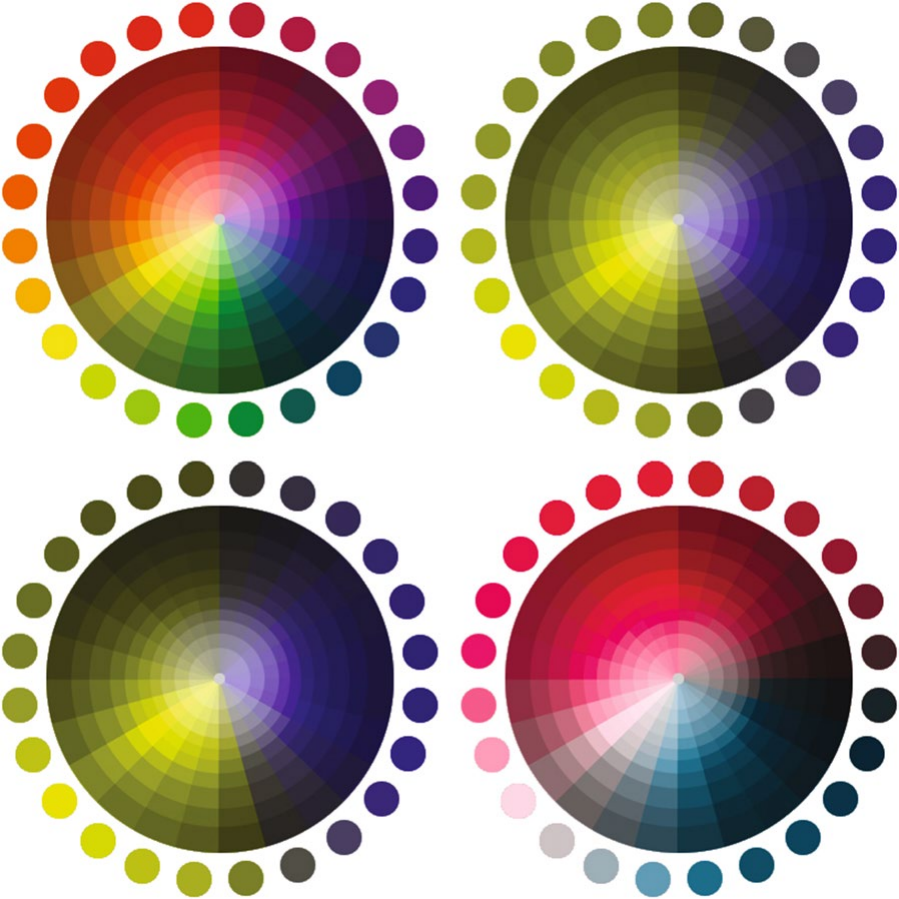
*Color wheel* and its simulation in 4 different profiles: non-disabled vision (*top-left*), deuteranopia (*top-right*), protanopia (*bottom-left*), and tritanopia (*bottom-right*)

This leads CVD users to a partial and biased perception of colors, as depicted in Fig. 2, where different types of deficiencies are simulated.

Access to information and services can be impaired by CVD. The main problems arise in reading textual information when the user interface is developed without taking color accessibility into account (Gradisar et al. 2007). Indeed, legibility is strongly related to the capability of discerning foreground and background colors, and there is general agreement in recognizing that higher luminance contrast improves legibility (Knoblauch et al. 1991). Recent studies (Zuffi et al. 2007; Gradisar et al. 2007) have confirmed that legibility is significantly affected by how colors are combined together, as difference between colors becomes more important when luminance contrast is lower. However, the effect of chromatic contrast is still unclear: high chromatic contrast between colors having similar luminance can still make the text legible, but no advantage has been found for low-vision reading (Legge et al. 1990).

Any attempt to address this issue should take into account that UI is generally designed by non-impaired users with few or no concern of CVD limitations. Therefore several methods have been developed in order to (1) support designers in the task of choosing colors or (2) to compensate deficiency by adapting the UI to CVD user needs. An overview of these methods is presented in “Appendix”.

Among the several proposals, a promising approach is based on looking at adaptation in terms of search, so that a color palette is optimized with respect to CVD user needs, but preserving the chromatic choices operated initially by the UI designer. For instance Ichikawa et al. (2003) make use of a genetic algorithm in order to optimize the contrast as perceived by impaired users, and preserving the image chromatism when a Web page is rendered.

Similarly, in previous work (Troiano et al. 2008b; Birtolo et al. 2009; Troiano and Birtolo 2014) we investigated and compared genetic algorithms in assisting the design of color accessible interfaces. UI designers choices and preferences (e.g., preserve the meaning of colors) can often conflict with the need of assuring high luminance contrast between colors. An algorithm can be employed to test different combinations of colors in attempt to best solve those conflicts. Genetic algorithms are able to face effectively this problem, as they are able to explore a large combinatorial search space, exploiting those solutions that reveal a good fitness to a set of (even conflicting) requirements. Indeed, using a genetic algorithm provides the following advantages:

- A large number of alternatives can be explored and used to support human creativity and decision-making
- A trade-off between conflicting criteria can be obtained by considering different quality attributes and design guidelines at the same time
- Designers can focus on more value-adding tasks, letting the algorithms to fine-tune their choices
- Interfaces can automatically accommodate impaired user needs

In particular, we prototyped two solutions based on (i) *standard genetic algorithm* (SGA) (Troiano et al. 2008b) and on *interactive genetic algorithm* (IGA) (Birtolo et al. 2009). More details can be found in “Appendix”. By analyzing both algorithms from a computational point of view (Troiano and Birtolo 2014), SGA and IGA showed the capability of converging towards highly fitted solutions. IGA proved to be feasible and advantageous when compared to non-interactive genetic algorithms due to its ability of capturing fitness attributes related to human perception, that cannot be caught by a mathematical model. However, (if possible) quantification of preferences still makes possible to reduce the subjectivity associated to them. In the attempt of performing an analysis that is more robust and independent from the human perception, we simulated the expected behavior by software. However, this was an experimental setting whose results attain to simulation, rather than real operating conditions. So we moved to an experimental setting aimed at testing solutions with real users, looking for a validation of expected benefits.

## Experimentation

We designed the experiments having in mind the question if it is possible to provide solutions comparable or better than those made by humans, in a shorter time. Although simple, such a question is not trivial due to combinatorial complexity of search and quality of solutions attaining human aesthetics and creativity. Experimentation was aimed at assessing the quality of adapted solutions from both a qualitative and an usability point of view, paying particular attention to color vision disorders, Deuteranopia in particular. Experiments were led at Research and Development Centre of Poste Italiane, Naples. They were organized in two stages: Experiment 1 and Experiment 2.

Experiment 1 was aimed at verifying if the automatic tools based on SGA and IGA were effective in supporting the UI design and if it was possible to discern solutions produced by tools from those produced manually. For this purpose we engaged 6 participants in performing a color adaptation with each of the 3 methods (manual, IGA and SGA) and we recorded the time to complete the task. After, for each method, we selected solutions which scored best for each method and we proposed them to a panel of other 14 participants who were asked to respond to a questionnaire aimed at assessing the solutions under different qualitative criteria. Among the questions also the request of scoring each solution and selecting which of them was made manually in their opinion. The participants were not chosen with any specific color vision disorder.

Experiment 2 was performed in order to verify if solutions produced along the previous experiment were actually improving accessibility and usability for CVD users. For this purpose, we considered the best automated solution selected by participants to Experiment 1 and compared to the manual solution in solving a memory and selection task by a panel of 20 CVD participants. Both experiments are within-subjects.

Below we provide experimentation the details regarding participants, equipment materials, experiment procedures and results.

### Participants

For the experimentation we enrolled 40 participants within employees of Poste Italiane. We enrolled the participants in two different phases. During the first phase (Experiment 1) we selected 20 participants. The average age of the participants of this first group was 37.7, ranging from 28 to 55 years old. Ishihara test was adopted for all participants in order to verify possible color vision deficiencies. Two participants confirmed to be color blind users.^4^ In particular both participants were affected by deuteranopia. The group was involved in assessing the quality of solutions provided algorithmically when compared to those produced manually.

For the second phase (Experiment2), we selected a group of 20 participants, whose average age was 33.4 years old, ranging from 26 to 61 years old. This group was composed by deuteranopes. The CVD was confirmed by a preliminary Ishihara test. Due to difficulties to select and involve an appropriate group of users, this required time to complete the experimentation. The group resulted being geographically and socially more heterogeneous, but all the participants were habit to use computers, during both working and free time. This group was involved in usability tests in order to assess real benefits obtained by adapted color palettes.

### Equipment and materials

We arranged a single-pc room in our Laboratory in conformance to the ISO 9241 standard. The ambient was neutral and light was sourced in the room by shielded lamps on the ceiling. Other light sources, such as those coming from windows, were curtained. The room illumination was below 300 lux. Attention was paid in order to avoid any glare or reflection on the monitor screen.

The experimental observations were carried out on Intel Pentium IV machine with 2 GB of RAM running Windows XP Professional Edition SP2 equipped with BenQ T720 LCD monitor, standard keyboard and optical mouse. The display inclination was $100^{\circ }$, with screen size of 17-inch, resolution of 1280*1024 pixels without interpolation and refresh rate of 75Hz. The monitor white point was set to D65 and the maximum luminance was 160 $\mathrm{cd}/\mathrm{m}^2$. The CIE XYZ of the LCD white point was (95.047, 100.000, 108.883).

**Fig. 3:**
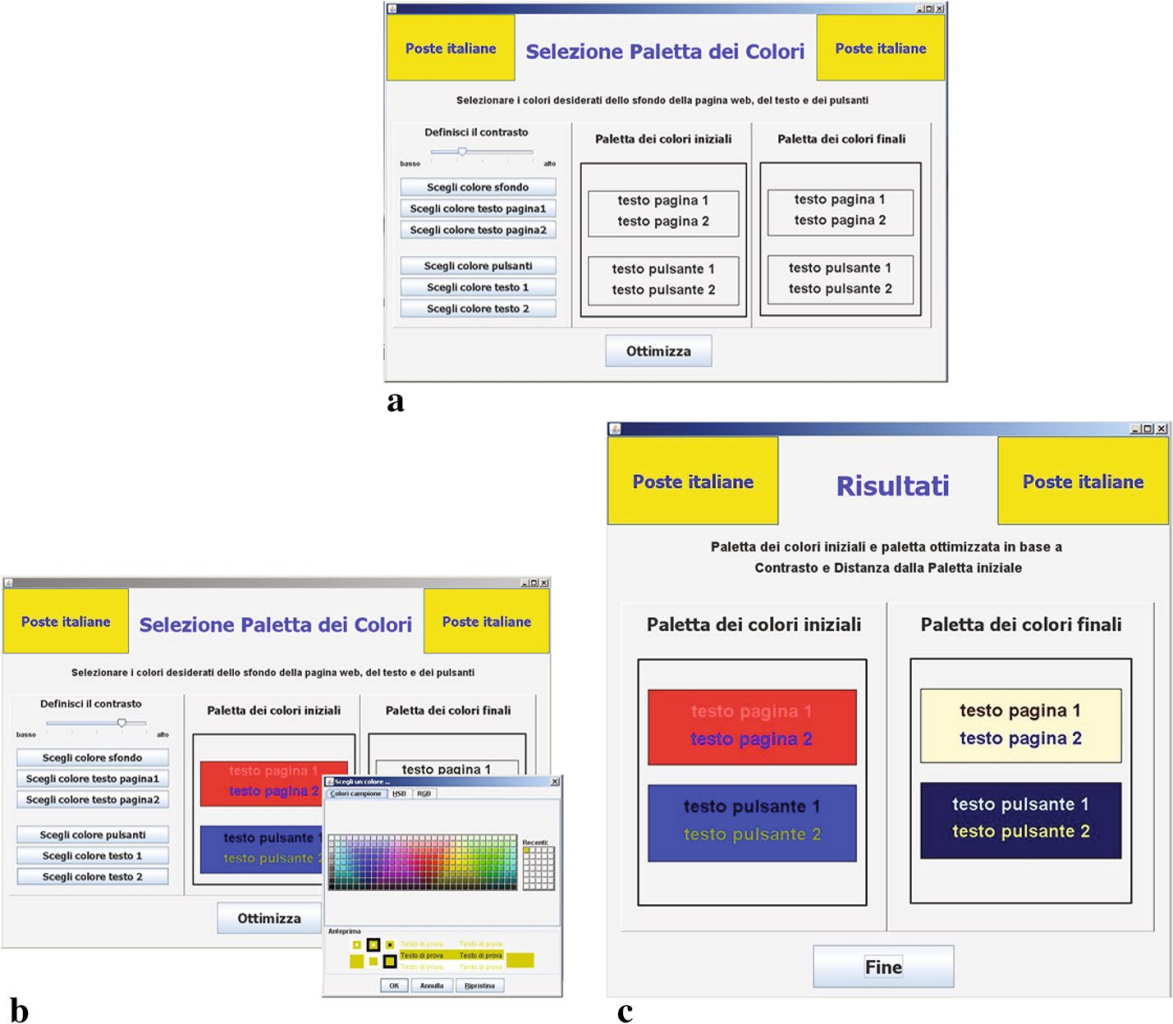
Tool used for experimentation. **a** Initialization: choosing contrast ratio required and initial colors. **b** Choosing foregrounds and backgrounds of the interface. **c** Results: Optimized colors

For experimentation, we implemented a tool by which the initial palette is specified (Fig. 3b) with relations between colors and the desired contrast ratio (Fig. 3a). The output is an optimized palette which preserves chromatic choices, but guarantees accessability at the same time (Fig. 3c).

The tools supports both IGA and SGA modes. In details, IGA mode entails an interactive selection of preferred solution so that the color preferences are expressed implicitly by awarding the best attractive color combinations, while SGA tool entails a totally automatic procedure where the preferences are explicitly defined and coded before the algorithm starts.

Evaluation of results was performed by the following free software: (1) *Colour Contrast Analyser* (version 2.0, WAT-C 2007), a tool for checking if fore-background color combinations provide good color visibility; (2) *ColorSelector* (version 5.1, Fujitsu Lmt. 2008), a Java Application aimed at evaluating whether fore-background combinations make the text readable to color blind users; (3) *ColorDoctor* (version 2.1, Fujitsu Lmt.) and *Color Tester* (Idea Futura), simulators able to check color accessibility according to W3C Recommendations.

### Preliminaries

All participants were briefly introduced to the aim of this study, they received an overview of the usability test procedure, and they gained access to equipment and software. The two groups received clear instructions about the task and how to use the available materials. In particular, participants to the first group involved in producing color schemes were trained on the design process in order to modify colors in a target web page and received an overview of the equipment and software, while the second group was trained on the usability test procedure. Both groups received a brief introduction to W3C color recommendations. Users were asked to assume a comfortable position such to guarantee a distance of about 50 cm from the display, in order to subtend a visual angle size of about $2.3^{\circ }(\mathrm{height}) \times 2.7^{\circ }(\mathrm{width})$, less than $4^{\circ }$ so the CIE1931 Standard Colorimetric Observer was used in calculations. The test stimuli pattern consisted of a full-screen desktop application. During the execution of both tasks the trainer assisted the participants and provided some technical help when needed.

### Experiment 1

#### Procedure

Participants were divided in two focus groups: 6 participants, with an appropriate skill in designing web pages and without any kind of CVD, were assigned to the first focus group. The remaining 14 participants were enrolled in the second group. The average age of the two groups was respectively 32.7 and 40.3 years old.

**Fig. 4:**
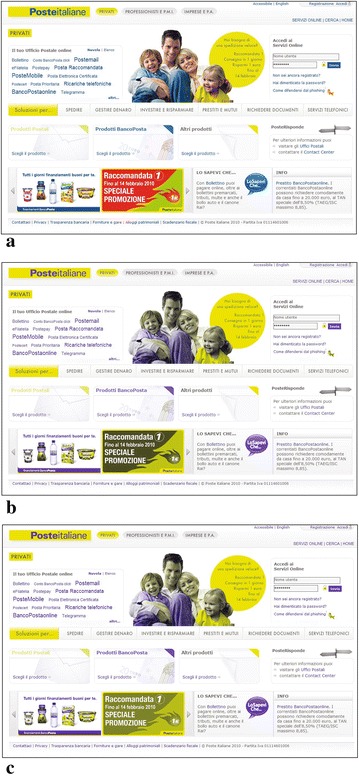
Initial web page. **a** Original web page. **b** Simulation of protanope vision. **c** Simulation of deuteranope vision

**Fig. 5:**
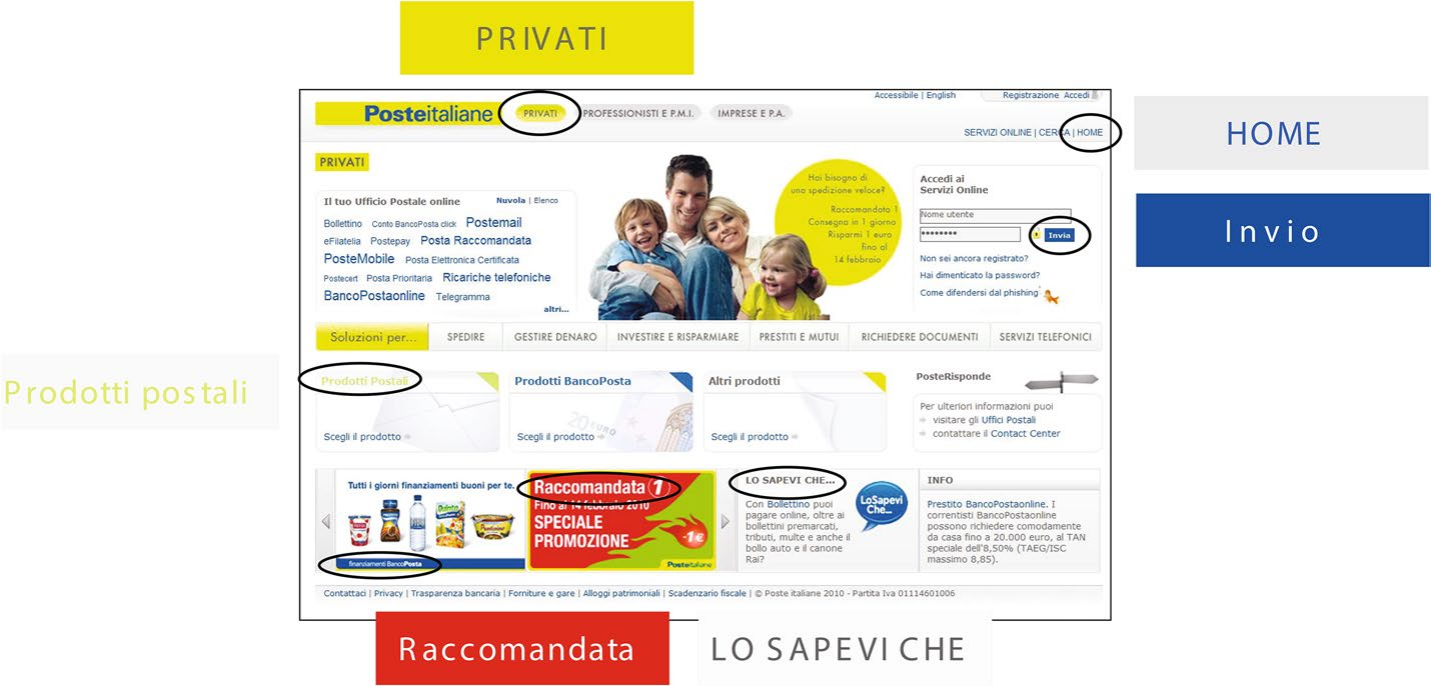
An example of accessibility issues expressed as pairs of foreground/background colors

The task of the first group was to upgrade a target page towards an accessible web page regarding the use of colors. As target we chose the page available at url http://www.poste.it/privati and depicted in Fig. 4. In Fig. 5, we highlights 6 color combinations that convey information, thus they should be made accessible to CVD users.

The initial page presented some accessibility issues, as summarized in Fig. 5. In particular some color combinations were very critical due their low contrast ratio. The task assigned to the designers was to modify the color in order to achieve an improved version of the web page. This task was performed by 3 methods:

1. the designers modified manually the colors, assisted by Colour Contrast Analyser and/or ColorSelector in measuring the contrast ratio;
2. solutions were automatically produced by SGA tool;
3. the designers were supported by IGA tool in selecting the combinations of colors that best fit his/her preferences and letting the algorithm to evolve solutions and guarantee accessibility.

**Fig. 6:**
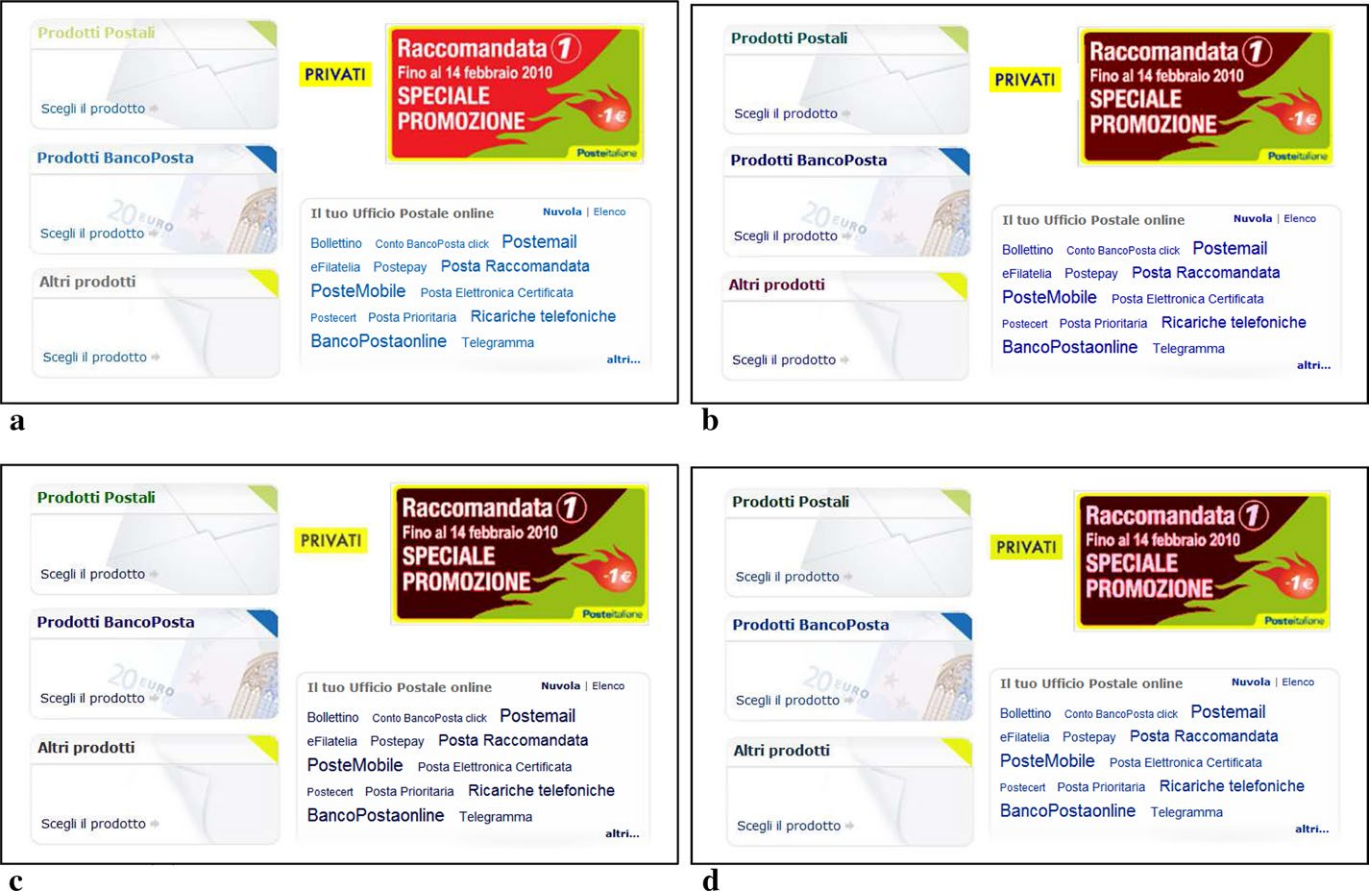
Color schemes after the first group of Experiment 1 completed the task. **a** Initial scheme. **b** Solution 1: Manual Procedure. **c** Solution 2: SGA Tool. **d** Solution 3: IGA Tool

Time-on-task records are reported in Table 2. Once the first group completed the task, we collected 6 solutions for each method, totally 18. In order to reduce the number of solutions proposed to the second focus group, for each method we chose the solution with the highest score (i.e., fitness value in the lexicon of evolutionary computing). Solutions provided are presented in Fig. 6. Then, the second group evaluated the proposed solutions. The method used to generate the solution was kept hidden to the group. The group evaluated individually the three different solutions by means of a questionnaire made of the following questions:

- *Question 1*: The selected elements satisfy aesthetics requirement.
  (Strongly disagree 1.2.3.4.5 Strongly agree)
- *Question 2*: Web page has a colors that make it especially appealing.
  (Strongly disagree 1.2.3.4.5 Strongly agree)
- *Question 3*: Contrast ratio between foreground and background is suitable.
  (Strongly disagree 1.2.3.4.5 Strongly agree)
- *Question 4*: Information is easy to read.
  (Very difficult 1.2.3.4.5 Very easy)
- *Question 5*: My overall impression of colors is
  (Very Negative 1.2.3.4.5 Very positive)
- *Question 6*: I think that the adaptation of color for ensuring accessibility is developed by
  (Human, Computer, I don’t know)
- *Question 7*: Solution Ordering from the worst to the best
  (Solution 1, Solution 2, Solution 3, Original Solution)

User group had to give a level of agreement to each question with a score ranging from 1 (Strongly disagree) to 5 (Strongly agree), except of the last two questions.

**Table 2:** Execution time expressed in seconds

Designer No.	Manual	IGA tool	SGA tool
1	825	370	210
2	720	305	231
3	580	325	164
4	610	390	192
5	490	233	82
6	535	246	110

#### Results

The first hypothesis we tested is if solutions provided by Humans (S1), IGA (S2) and SGA (S3) are perceived as equivalent. A Kruskal–Wallis test on answers given by the 14 respondents provided an affirmative conclusion, as reported in Table 3 (tabled chi-squared 5.9915).

**Table 3:** Kruskal–Wallis statistics

Question No.	p values	KW chi-squared	Mean Score
1	0.9718	0.0571	3.238
2	0.7994	0.4477	3.048
3	0.96	0.0815	3.857
4	0.3722	1.9768	4.286
5	0.9674	0.0663	3.333

Obviously, the same conclusion can be reached by a pairwise (two-sided) Wilcoxon rank-sum test to the first five questions, whose result is reported in Table 4, even when no correction due to multiple comparisons is performed (e.g., Bonferroni, Holm, Hommel, Benjamini-Hochberg, etc.).

**Table 4:** Wilcoxon rank-sum test: p values

Question No.	S1 versus S2	S1 versus S3	S2 versus S3
1	0.2402	0.484	0.5716
2	0.2815	1	0.5182
3	0.9168	0.8241	1
4	0.0969	0.7897	0.1581
5	0.887	0.5654	0.4537

In addition, answers given to the Question 6 entail the group was not able to recognize S2 as generated by machine (57 % said by human). In general only 14.3 % was able to correctly distinguish this solution from the others.

In the focus group we extracted 2 different sub-groups: CVD users (2, affected by Deuteranopia) and Seniors (2, over 50 years old). As described before, each of them evaluated quality of solutions by answering the questionnaire. The question arisen here is to test if these users perceived the quality of the three solutions as different or not. Due to exiguity of the two groups we performed a comparison by performing a Wilcoxon rank-sum test paired on answers to questions in a row given by the two CVD users and the two Seniors for the different solutions, in order to check if answers were able to support a statistical difference facing S1 versus S2, S2 versus S3 and S1 versus S3.^5^ Looking at p values (Table 5, assuming p values <0.05 to reject test null hypothesis $H_0$, while p values >0.50 to accept $H_0$), we can state that (1) according to CVD users the 3 solutions presented some qualitative differences, preferring S3 as confirmed by answers to Question 7 which put S3 at the first position, while (2) answers provided by elder users did not entail significant differences. We interviewed the CVD users in order to understand why they preferred S3 over the others. They affirmed that solution was more appealing than others and colors appeared in general more pleasant.

**Table 5:** Pairwise Wilcoxon test: p values

User	S1 versus S2	S1 versus S3	S2 versus S3
Deuteranopes	0.0035	0.07186	0.0046
Seniors	0.7656	0.5877	0.2330

**Fig. 7:**
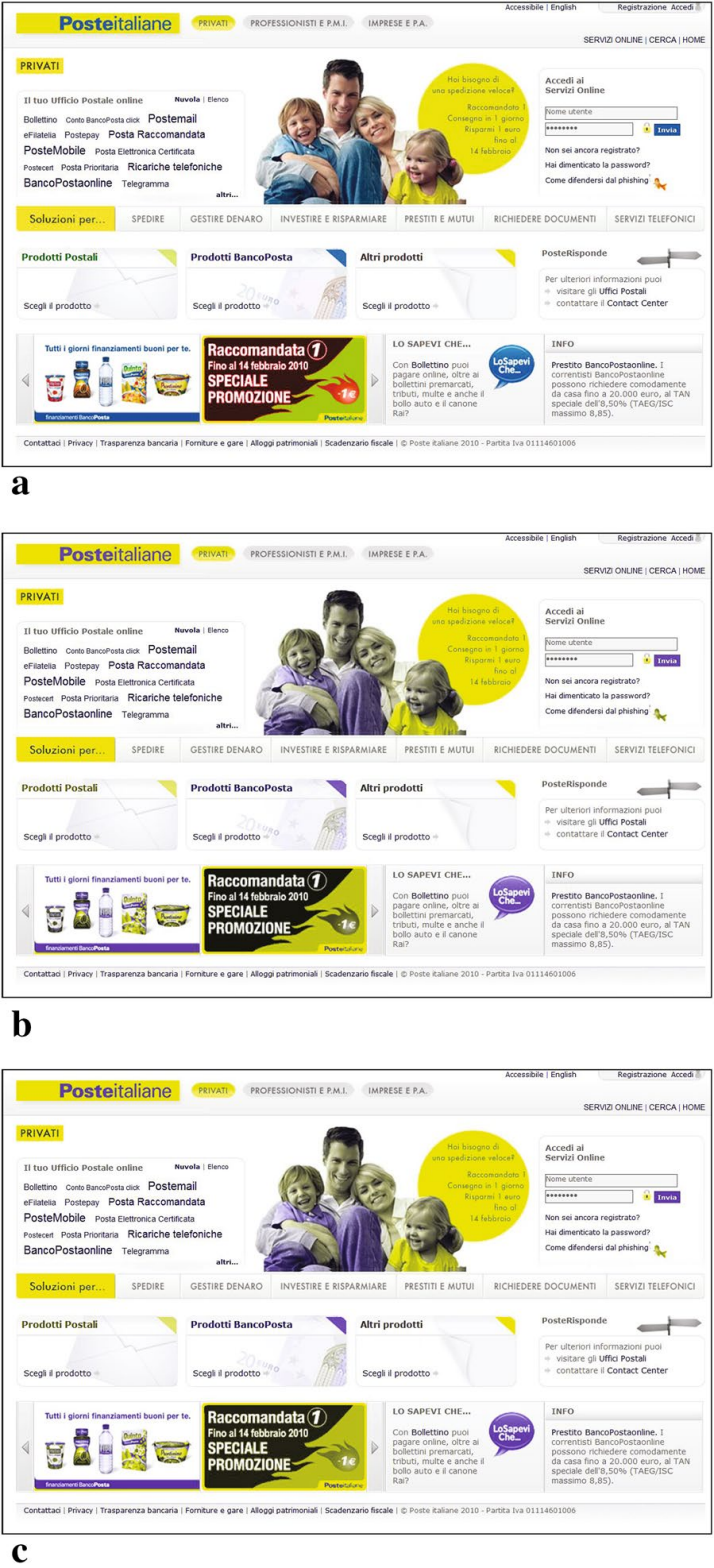
Resulting Web Page. **a** Non-CVD vision. **b** Simulation of protanope vision. **c** Simulation of deuteranope vision

Looking at answers to Question 7, 42.9 % selected S3 as the best one, while nobody indicated it as the worst one, whilst 71.4 % indicated the original color combination as the worst. Figure 7 reports the page modified according to scheme provided by S3 (IGA) and how it is perceived by CVD users.

Searching for a solution comparable to those made by humans could be of lesser interest if not obtained in lesser time. However, time-on-task reported in Table 2 outlines designers in the first group consistently experienced a shorter time in building a solution. Indeed, the manual method entails in all cases a higher execution time, while the automatic method provide a significant time saving. Higher time is justified by additional selection time with IGA and checking the contrast ratio in the manual approach, both not required by SGA.

### Experiment 2

#### Procedure

The aim of Experiment 2 was to test solutions from a usability point of view. Two solutions were taken into account: specifically, the solution provided manually and the solution generated by means of IGA (see Fig. 6). Solutions are submitted to the participants by means of (1) Selection Test Tool, and (2) Memory Test Tool.

The task implemented by Selection Test Tool consisted in finding a word (on the left) among a set of 20 words, displayed on the right side using a color combination. In order to focus the test on the effect of colors in recognizing the right word, the selection had place among a set of words with a similar typing. Selection had place by clicking on the corresponding word. Time elapsed from showing and selecting the word was recorded. In case of wrong selection, the test goes ahead to the next word. So that, time for both correct and wrong selections was recorded. Test is iterated along 5 words, per user and per color solution. In order to reduce user fatigue a break of 5 minutes was given between the two solutions being tested.

Instead, the task implemented by Memory Test Tool consisted in reading a sequence of 3 words, randomly chosen among a dictionary of 20 words, and asking the participant to memorize the sequence, then to answer if a word appeared or not in the sequence.

The test performed three sequences of words per user and per solution. Again, in order to make experiment more focused on how color combinations can affect short-term memory, the set of words was made of similar typing. For both the applications, we adopt time-on-task metrics and correctness metrics.

#### Results

We aimed at investigating from usability point of view, the difference between solutions provided by humans (solution *H*) and solution adapted by genetic algorithms (solution *G*), when submitted to deuteranopes.

During the memory test, we assumed that a respondent was able to remember words appearing in sequence, so to recognize if specific word was displayed or not.

We defined *correct memorization rate* (CMR) as the fraction of correct answers over the total number of questions. CMR for human (*H*) and algorithmic (*G*) solution was 0.867 and 0.933 respectively as shown in Table 6. Analyzing in depth the results, we note that the number of respondents giving at least a wrong answer, was 7 in the case of *H* and 4 in the case of *G*. This leads to the conclusion that words submitted with the color scheme provided by IGA were easier to memorize.

**Table 6:** Memorization task and CMR for the two solutions after 60 trials (20 users)

Solutions	Answer time (s)		CMR
	Average	SD	
H	3.016	1.798	0.867
G	2.535	2.156	0.933

Moreover, we compared the response time by means of a Wilcoxon paired test. Looking at p value (0.02482), we can observe a statistical difference between solutions (in particular a shorter answer time in case of *G*) with a confidence ${\ge}95\;\%$.

There is no significant difference, instead, if we consider the selection test. Similarly to CMR, we define *correct selection rate* (CSR) as the number of correct selections over the overall number of selections. CSR is comparable in the case of both solutions. It is 0.98 for *H* and 0.99 for *G*, as reported in Table 7. Investigating the response time by Wilcoxon paired test and looking at p value (0.896), we can state that there is no statistical difference in response time between *H* and *G* (strong accept at p value 0.5). Thus participants took a similar time in recognizing and selecting the proposed word.

**Table 7:** Selection task and CSR for the two solutions after 100 trials (20 users)

Solutions	Selection time (s)		CSR
	Average	SD	
H	3.897	1.848	0.98
G	3.893	1.924	0.99

The latter findings can be justified by the way a word is recognized within a set. Indeed, the task of picking a word in a set is mostly influenced by the recognition of specific patterns of letters within a word, more than the whole word itself. This was confirmed by respondents interviewed after performing the test.

### Limitations

The lack of evidence in Experiment 1 in rejecting the hypothesis that a statistical difference stands out from answers to the questionnaire can only marginally support the assumption that no difference will be reproduced in any further experiment. This makes difficult to draw conclusions in general terms. In addition, the standard deviation in answers varies between 0.663 and 1.342. Therefore, improving the statistical power would require to involve a larger group of participants. Similar considerations can be made for Experiment 2 in testing the response time.

## Conclusions

Color accessibility represents a relevant barrier for CVD users in gaining access to the Information Society. Since user interface is generally conceived by non-CVD designers for non-CVD users, there is a need to support the choice of alternative color scheme able to better address color vision disorders. So far, the effort has been focused on building tools able to measure the luminance contrast ratio and to simulate CVD vision, leaving the designer to choose among alternatives. However the large number of combinations can make such a task time demanding.

The space of color palettes can be explored searching for a solution able to provide a positive trade-off between aesthetics and accessibility. In particular, genetic algorithms driven by a mix of color metrics and designer preferences can be employed for such a search.

The study presented in this paper attempted to provide an answer to the question if solutions that proved to work algorithmically, are also effective when translated to practice. Results from experimentation have been proving that GA search based approach is feasible, both when designer preferences are explicit and implicit. In the first case search is driven only by color contrast ratios and distance from the original color pattern. In the second case, the algorithm iteratively combines metrics and human subjective feedback, leaving the designer free to move towards a different color scheme along the evolution process. In both cases, time-to-task proved to be largely smaller than the manual approach, providing solutions comparable and sometimes preferable to those obtained with no support in searching color alternatives.

Experimental results from this study make possible to outline the following conclusions:

- Automatic support offered an effective support by allowing a reduced time-on-task with respect to a purely manual color adaptation (Experiment 1)
- Solutions proposed by genetic algorithms is not perceived of lower quality (Experiment 1)
- Adaptation provided by genetic algorithms is beneficial in order to make the UI accessible to CVD users (Experiment 2)

Experimentation might present some weakness. Due to time constraints, the experiments were performed on single page, so that its layout might have influenced the measurement. However, since the study is comparative, this should not have affected the conclusions. The initial page has a color scheme where white is predominant over the other colors. This could make difficult to capture differences in solutions, thus affecting the answers collected by the questionnaire. However this is a common situation in most web pages. We attempted to limit this effect by providing summary pictures as those depicted in Fig. 6. Another point regards the composition of the second focus group in Experiment 1. Among them we had only two known CVD users. However the page was chosen according to the deficiency. A posterior interview confirmed that user preferences were driven by usability issues more than a personal sense of aesthetics. In addition, the number of participants was not large enough to study if there is a correlation between preferred solutions and the age of respondents, as we did not have a significant representation for each age class. Indeed, age can affect how colors are perceived although Ishihara test is passed. Time-on-task could be influenced by the usability of UI, so that experiment outcomes should be consider only for comparing the three different approaches. Finally, colors attains to cultural background, and the focus groups were made of the same ethnic group. This represents a limitation to universally extend the experimental results. An additional weakness related to Experiment 2 is related to confidence with words. Although, we chose common words within the Italian dictionary, we did not measure this aspect. Finally, we did not measure the severity of the disease as this attains to medical practice and not allowed by internal policies.

Besides these aspects, conclusions reached by this study offer several points of confirmation (although preliminary) to the validity of pursuing CVD adaptation by means of tools able to free the UI designers from considering accessibility constraints in choosing a color scheme, letting the tools to reach an optimal trade-off. We hope this will foster further research in this direction.

## Figures and Tables

**Fig. 8:**
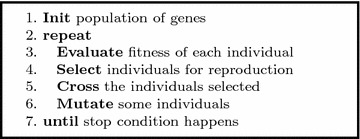
Simple genetic algorithm outline

**Fig. 9:**
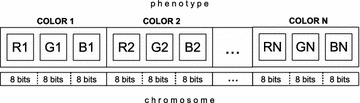
Chromosome structure and phenotype mapping

**Fig. 10:**
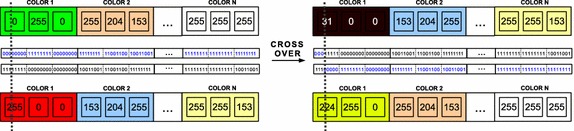
Crossover

**Fig. 11:**
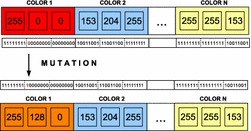
Mutation

**Fig. 12:**
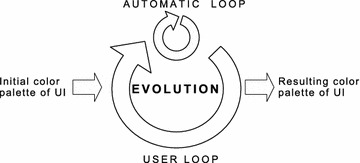
Interactive evolution

## Appendix: Brief overview of the field

In this section we provide a brief overview of the field in order to make the reader informed of developments in CVD modeling and mitigation techniques, with a specific focus on algorithms employed in the experimentation.

### Color models

Color models define spaces in which colors are described as tuples of numbers (typically three or four values), called *color components*. RGB and CMYK are very well known color models. In RGB, the three primary components *R* = Red, *G* = Green, *B* = Blue provide the basis for getting colors as an additive combination of them. Vice versa in CMYK, primary components are *C* = Cyan, *M* = Magenta, *Y* = Yellow, *K* = blacK, and colors are obtained by a subtractive aggregation of them. Both RGB and CMYK models suit well in describing how to produce or print colors through devices, but they are not related to the perception of colors.

Other models are closer to the way colors are perceived. The French *Commission Internationale de l’Elcairage* (CIE) introduced the XYZ model. The *X*, *Y*, and *Z*, are obtained by integrating the spectral energy emitted by the light source multiplied by the three CIE Standard Observer functions. Although introduced in 1931, this model is still popular among practitioners as pivotal in converting colors from one model to another. However, XYZ do not represent the response of cones to at short, middle and long wavelengths.

In 1976, the CIELab model has been proposed in the attempt of better describing the human perception. In this case, components are ${L^*}$ that is a measure of color luminance, $a^*$ being its position of red/magenta and green, and $b^*$ its position between yellow and blue. When $L^*=0$ we get black, while $L^* = 100$ corresponds to white. The model has been designed so that uniform changes in the CIELab coordinates correspond to uniform changes in the perceived colors. This property makes CIELab suitable for measuring the perceptual distance between colors by means of the Euclidian distance between points in $L^* \times a^* \times b^*$ color space, as it provides a measure of both hue and density changes. Another characteristic is that a given triplet $(L^*,a^*,b^*)$ is able to refer the same color independently from the source, making CIELab more objective and device-independent than other models.

Generally, color spaces (i.e., color models) are homeomorphic: there exist formulas able to move from a color representation in one space into an equivalent representation in another space, and vice versa.

### Methods for simulating and compensating CVD

CVD simulation methods can be dated back to the early 19th century, when Goethe painted a water-color landscape intended to simulate the perception of scene got by a tritanope observer. Today, it is possible to benefit of scientific advances in the studies of human vision and use digital image processing in order to experience color vision deficiencies.

To simulate dichromatic vision, Brettel et al. (1997) designed an algorithm which works in the LMS wavelength color space, performing a projection according to the type of dichromatism. The algorithm is made of three steps:

- LMS values are derived from RGB coordinates
- Colors are projected in the LMS space in order to simulate the deficiency
- New RGB color values are obtained by converting the projected LMS coordinates

Viénot (1999) proposed to use projection in the LMS space to build replacement color-maps of the standard 256 colors (including 216 colors that are common to many applications in Microsoft Windows and Macintosh operating systems) as means for a designer to simulate how an application user interface is perceived by protanopes and deuteranopes. His method, still based on projections in the LMS color space, is made of seven steps. An example of color simulation has been provided in Fig. 2. In the following section we briefly outline this method.

### Simulating CVD

In order to compute the contrast ratio as perceived by CVD users, we are in need of converting colors. According to the model presented in Viénot et al. (1999), color coding triplets (*R*, *G*, *B*) (generally expressed as 8-bit integer values) can be transformed into photometric quantities $R_1$, $G_1$, $B_1$, corresponding in turn to the red, green and blue component. Then, reduced colors in the gamut of display are obtained by rescaling photometric quantities to $R_2$, $G_2$, $B_2$.

According to Smith and Pokorny (1975), and following the method outlined by Viénot et al. (1999), we make use of the following equation.

1 $$\begin{aligned} \begin{aligned} \left( {\begin{array}{*{20}c} L \\ M \\ S \\ \end{array} } \right)&= \left( {RGB\_to\_LMS} \right) \left( {\begin{array}{*{20}c} {R_2 } \\ {G_2 } \\ {B_2 } \\ \end{array} } \right) \\&= \left( {\begin{array}{*{20}c} {\text {17}\text {.8824}} &{} {\text {43}\text {.5161}} &{} {\text {4}\text {.11935}} \\ {\text {3}\text {.45565}} &{} {\text {27}\text {.1554}} &{} {\text {3}\text {.86714}} \\ {\text {0}\text {.0299566}} &{} {\text {0}\text {.184309}} &{} {\text {1}\text {.46709}} \\ \end{array} } \right) \left( {\begin{array}{*{20}c} {R_2 } \\ {G_2 } \\ {B_2 } \\ \end{array}}\right) \end{aligned} \end{aligned}$$

that is the mapping of RGB stimuli to LMS wavelength space attaining the standard response of cones.

In this method, we perform a reduction of the trichromatic colors to the dichromatic domain, keeping the light absorption performed by S and M photopigments for protanopes, and L and S for photopigments. Therefore, the trichromatic color space can be mapped over a reduced dichromatic plane, due to the remaining two photopigments, whose equation is

2 $$\begin{aligned} \alpha L + \beta M + \gamma S + \delta = 0 \end{aligned}$$

where $\delta = 0$ (i.e., the origin belongs to the plane) as the black, entailing S = M = L = 0, is still perceived unchanged. Also the white point $(S_W,M_W,L_W)$ and the blue primary $(S_B,M_B,L_B$) are perceived unaltered, thus we get

3 $$\begin{aligned} \alpha & = M_W S_B - M_B S_W \nonumber \\ \beta &= S_W L_B - S_B L_W \nonumber \\ \gamma &= L_W M_B - L_B M_W \end{aligned}$$

The projection on protanope plane entails

4 $$\begin{aligned} L_p & = -(\beta M + \gamma S) / \alpha \nonumber \\ M_p & = M \nonumber \\ S_{p} & = S \end{aligned}$$

while on deuteranope plane we get

5 $$\begin{aligned} L_d & = L \nonumber \\ M_d & = -(\alpha L + \gamma S) / \beta \nonumber \\ S_d & = S \end{aligned}$$

In particular for protanopes, we have^6^

6 $$\begin{aligned} \left( {\begin{array}{*{20}c} {L_p}\\ {M_p}\\ {S_p}\\ \end{array}}\right) = \left( {\begin{array}{*{20}c} 0 &{} {\text {2}\text {.02344}} &{} -{\text{2}\text {.52581}} \\ 0 &{} 1 &{} 0\\ 0 &{} 0 &{} 1\\ \end{array}}\right) \left( {\begin{array}{*{20}c} L\\ M\\ S\\ \end{array}}\right) \end{aligned}$$

while for deuteranopes

7 $$\begin{aligned} \left( {\begin{array}{*{20}c} {L_d}\\ {M_d}\\ {S_d}\\ \end{array}}\right) = \left( {\begin{array}{*{20}c} 1 &{} \text {0} &{} \text {0} \\ {\text {0}\text {.494207}} &{} 0 &{} {\text {1}\text {.24827}} \\ 0 &{} 0 &{} 1 \\ \end{array}}\right) \left( {\begin{array}{*{20}c} L \\ M \\ S \\ \end{array}}\right) \end{aligned}$$

Transformation of $$L_d$$$$M_d$$$$S_d$$ or $$L_p$$$$M_p$$$$S_p$$ to RGB is obtained using the inverse matrix of

8 $$\begin{aligned} \left( {\begin{array}{*{20}c} {R_d } \\ {G_d } \\ {B_d } \\ \end{array}}\right) = \left( {RGB\_to\_LMS} \right) ^{ - 1} \left( {\begin{array}{*{20}c} {L_d}\\ {M_d}\\ {S_d}\\ \end{array}}\right) \end{aligned}$$

The contrast ratio as perceived by CVD users can be thus obtained by the altered components $R_d$, $G_d$ and $B_d$.

In the past years, several automatic adaptation and recoloring algorithms have been proposed in order to accommodate the content for CVD users. For instance, Ou-Yang and Huang (2007) suggest an analytic framework to analyze the relation between brightness and color gamut, thus to control the saturation and hue of confused colors in order to improve discernibility.

Flatla et al. (2012), Flatla and Gutwin (2012) propose color differentiation models, named Situation-Specific Models (SSMs), designed to fit the impaired user needs and the environmental context with respect to which the model is calibrated and used, so that the recolored images attempt to reflect accurately the color vision abilities of the user in a particular environment.

Other solutions attempts to recolor images by minimizing the distortion of color features, such as the hue, brightness and contrast This is the case of Color-via-Pattern (CvP), an initial work proposed by Herbst and Brinkman (2014) which preserves the perceived brightness, or Mereuţă et al. (2012) solution, which preserves the color contrast for dichromat users.

Color remapping is generally performed offline. But it can also be also, such as in the proposal of Chroma based on Google glasses proposed by Tanuwidjaja et al. (2014), smart glasses proposed by Popleteev et al. (2015), or ColorBless, based on stereoscopic 3D displays proposed by Hau Chua et al. (2015).

Recently, instead of working on the color map, Flatla et al. (2015) proposed to alter the image texture in order to include information able to compensate the loss of information produced by color vision deficiency.

### Adaptation as search

Instead of providing palettes and correspondences, an alternative approach consists in assuming the problem of finding a suitable combination of colors as an optimum search. In this case the search space is made of a whole color space model (e.g. RGB, CMYK, CIELab, etc.) or a reduced one. Candidate solutions are specific combination of colors (i.e., color palettes), each providing a different degree of color accessibility. Therefore, the goal is to find the solution that best fits the CVD user needs.

Jefferson and Harvey (2006, 2007) formulate the optimization problem in order to find the solution that maximizes the transfer of the chromatic information from the defective cone to the others. Their algorithm is made of four steps:

1. A subset of key colors is picked from the source image
2. Color and brightness differences between key colors are computed
3. Optimization to search an adaptation of colors for the dichromatic user is performed
4. Remaining colors are varied by using an inverse-distance weighted interpolation of transformed key colors

Rasche et al. (2005) face the problem of finding the gray-scale transformation that maximally preserve information for CVD users. Huang et al. (2007) present a recoloring method aimed at maximally discerning colors (details) while keeping the recolored image as much natural as possible (naturalness). The objective function is based on the linear combination of the two error functions weighted by a user-specified parameter, in order to adjust the trade-off between details and naturalness.

Recent developments make use of use meta-heuristic and evolutionary techniques to effectively explore the color space. Ichikawa et al. (2003) investigated how to improve the contrast offered by Web pages as perceived by impaired users, but still preserving the image chromaticism. To do this, they implemented a genetic algorithm and used CVD simulation for assessing the explored solutions. In particular, the page is decomposed in a hierarchy of colored regions. The spatial relation between colors determine which pairs to modify. The genetic algorithm is used in order to find a combination of colors which accommodate contrast requirement but that minimize the distance from the original scheme. This result have been confirmed in our previous work (Troiano et al. 2008b). Therefore, from both studies, it emerges the possibility that designers can be supported by genetic algorithms in choosing a palette variation able to ensure the accessibility of contents. Following a different direction, multi-objective optimization based on algorithms NSGA-II (Deb et al. 2002) and NSGA-III (Deb and Jain 2014; Jain and Deb 2014) has been experimented to adapt web pages in accordance to a set of preferences (Bonavero et al. 2015).

As already described, our attention focused on how to support the design of color accessible interfaces by a generative approach based on genetic algorithms (Troiano et al. 2008b; Birtolo et al. 2009; Troiano and Birtolo 2014). When choosing an appropriate color palette accessible to CVD users, we should: (1) consider a significant lightness difference between foreground and background colors, even when they differ in saturation or hue; (2) keep into account color wheel (see Fig. 2), avoiding to face light colors from the bottom half to dark colors in the top half; (3) avoid contrasting values from adjacent sections of the hue wheel, especially if the color does not contrast sharply in lightness (Arditi and Knoblauch 1996). In problems of practical interest, the number of pairs to be considered significantly increases.

As first step, we experimented the application of a standard genetic algorithm (SGA) (Troiano et al. 2008b) as baseline. The optimization objective was to select a palette able to improve the luminance contrast between colors, but still preserving the original chromatic setting. The structure of the algorithm is outlined in Fig. 8.

In addressing the problem, we start with an initial palette and a given contiguity relation between colors, e.g., such as between background and foreground colors. In order to formalize our problem in a GA domain, we encoded the palette in the chromosome as depicted in Fig. 9. In particular, the chromosome is a bit string, assuming 24 bits (8 bits per component) for coding each color in the RGB space.

Individuals represent color combinations, i.e., palettes. Those belonging to the initial population have been built as variations of the original color scheme. Genetic operators support GA processing by driving the evolution of solutions. *Selection* allows to promote those solution that best match criteria of optimality, represented by a score given by *fitness function* which trades off between design preference compliance and accessibility.

The *Crossover* operation swaps some part of genetic code string between parents in order to produce offsprings, emulating the crossover of genes in nature, so that descendants inherit characteristics from both parents. By this way, solutions are recombined in the hope that better combinations may emerge. As depicted in Fig. 10, crossover performs a color scheme mix at different levels. Bits of the RGB component at the location where the cross point has place are swapped. In addition, the remaining components of the color are mixed. Finally the colors on one side of the cross point are preserved while the colors on the other side are swapped between the two schemes involved.

The *Mutation* operation alters the value (i.e., allele) of some genes at very low rate, in order to escape from local optima. Figure 11 outlines how this affects the color scheme. By altering one bit at time, we produce a local variation at single RGB component. This allows to vary the color palette without heavily affecting its scheme.

In order the problem to be formalized as an optimum search we need to define metrics able to quantify differences between colors. The difference can be measured as distance between colors in a given color space. Since CIELab is the most appropriate for this purpose (as that is closer to human perception) we adopted definition given by CIE76 model, that is basically a measure of hue and density difference. In this space, colors at maximum distance $\Delta E_{\max }$ are green and blue.^7^

Since colors are not fully perceived in their spectrum by CVD users, also the difference in lightness, hue and saturations between them are altered, limiting the ability of discerning some combinations. More specifically, it is the contrast between colors that makes possible to distinguish contiguous regions in images. Therefore, we need to add a second metric able to quantify the contrast of colors. Contrast ratio ranges from 1:1 to 21:1. WCAG 2.0 assumes 7:1 as lower bound for accessibility (4.5:1 in the case of larger-scale text or images).

Optimization is performed by considering a fitness function of color distances and contrast ratios. We expect the fitness to improve by decreasing the distance and by increasing the contrast ratio. The *building block hypothesis* (Goldberg 1989) provides the theoretical framework able to explain the convergence of GAs (SGAs in particular) towards an optimal solution, so that the population evolves improving its average fitness generation by generation.

SGA makes an explicit use of preferences, so that preserving the meaning of colors, meeting harmony rules and following other aesthetic canons, should be coded and quantified in order to include both color metrics and preference scores into the fitness value. In other terms, the compliance to design preferences has to be quantified by means of some model able to score a color scheme against the set of designer preferences. The resulting score is combined to the score assigned to the scheme according to color metrics.

However, in some cases, preferences cannot be made explicit by any given model. In this case, the UI designer should be inquired in order to collect a feedback able to promote solutions that best fit his/her implicit preferences. Indeed, choosing a particular solution rather than others entails an implicit predilection in the scheme. In such cases, it is still possible to drive the color scheme evolution using the user feedback as a proxy.

Involving users in the evolutionary process can provide a valuable contribution, as preferences cannot always be made explicit. Human cognition can be used to solve conflicts arising between criteria along the design process. In addition, some color usability aspects may not be taken into the account at the initial color selection. Interactive genetic algorithms (IGA), as proposed by Takagi (2001), offer a meaningful approach in order to integrate human and artificial intelligence, especially when applied to artistic domains such as music and design. Some authors experimented IGAs in supporting interface design (Quiroz et al. 2007, 2009; Oliver et al. 2002; Banerjee et al. 2008). In these works, the user drives the IGA evolution of Web page features by selecting preferred solutions. Indeed, an IGA combines the power of human subjective evaluation with the generative optimization offered by evolutionary computing, so that the evolution is given by two loops. The inner loop (i.e.,*automatic loop*) aims at screening a set of alternatives searching for potential solutions. From those, a reduced set of alternatives is presented to the user and evaluated (i.e.,*user loop*), in the attempt of capturing user preferences and use them to drive the next evolution, as depicted in Fig. 12.

It is not feasible to ask the user to give feedback on each solution, because of the large number of alternatives being generated automatically by the inner loop. A smaller set of solutions has to be selected in order to limit the user fatigue when in collecting his/her feedback. Evaluating a large number of solutions can be tedious, demanding user attention for long periods. According to Llorà et al. (2005), user fatigue is to be avoided in order to produce high quality results.

Even inquiring the user to provide feedback every a given number of generations, the large collection of individuals produced by the algorithm does not make feasible a full assessment by the user. We need a different strategy to pursue: we can assess alternatives (i.e., individuals in the GA lexicon) indirectly by comparing them against few samples used to collect the user feedback. Those samples are chosen as qualified representatives of the whole population. So, the user can focus to only few but representative individuals, and a larger number of solutions can be explored and evolved.

## Footnotes

1 Is color blindness a disability? In many western countries (Italy included), color blindness is not considered a defect. In other countries, it is considered a problem that might exclude the access to some careers, e.g., in Japan, while in other countries people affected by color vision disorders are not allowed to drive. See www.colourblindawareness.org for more details. We share the idea that color vision disorders are source of impairment as they limit the access to the Information Society.

2 The Disability Rights Commission is an independent body established by the UK Parliament in 2000 aimed at stopping discrimination and promoting equality of opportunity for people with disabilities.

3 Data are public available at the Colour & Vision Research Laboratory of University College London (www.cvrl.org).

4 We did not use Ishihara test for diagnosis, as this attains to medical practice, but only as confirmation to information provided by the users.

5 The test is still within subjects, but instead of analyzing the answers given to each individual question by the different respondents, we assumed the sequence of 10 answers given by the two respondents as proxies of differences between the 3 solutions.
